# Supplementary material for: MetaRibo-Seq measures translation in microbiomes
Source: Nat Commun. 2020 Jun 29;11:3268. doi: 10.1038/s41467-020-17081-z (PMC7324362; doi:10.1038/s41467-020-17081-z)
Supplement: Supplementary file 10 — Supplementary Data 7 [file 41467_2020_17081_MOESM10_ESM.zip › File2/Confidence_High_Taxonomy/333149_out.krona.html]

Javascript must be enabled to view this page.

members
magnitude
magnitudeUnassigned
count
unassigned
taxon
rank

333149\_out

24

24
superkingdom
2

976
1
phylum

200643
1
class

171549
order
1

171550
1
family

genus
1
239759

species

SRS1041145\_contig\_number\_24307
1
214856

phylum
1
201174

84998
class
1

84999
1
order

84107
family
1

genus
1
102106


SRS078419\_contig\_number\_18800
species
1
74426

phylum
22
1239

13
class
186801

186802
13
order

172733
species

SRS011239\_contig\_number\_5190SRS013965\_contig\_number\_20498SRS045826\_contig\_number\_17432SRS049995\_contig\_number\_5802SRS053356\_contig\_number\_35097SRS054352\_contig\_number\_14556SRS055017\_contig\_number\_38411SRS078176\_contig\_number\_12565SRS142503\_contig\_number\_33915SRS143417\_contig\_number\_25521SRS893172\_contig\_number\_14510SRS893187\_contig\_number\_16568SRS893383\_contig\_number\_30609
13

1263003
9

SRS013521\_contig\_number\_11449SRS023914\_contig\_number\_26293SRS052697\_contig\_number\_40826SRS075773\_contig\_number\_26800SRS078242\_contig\_number\_28966SRS1041157\_contig\_number\_contig-100\_913.35284SRS104400\_contig\_number\_24722SRS148424\_contig\_number\_4271SRS971276\_contig\_number\_19653
species
